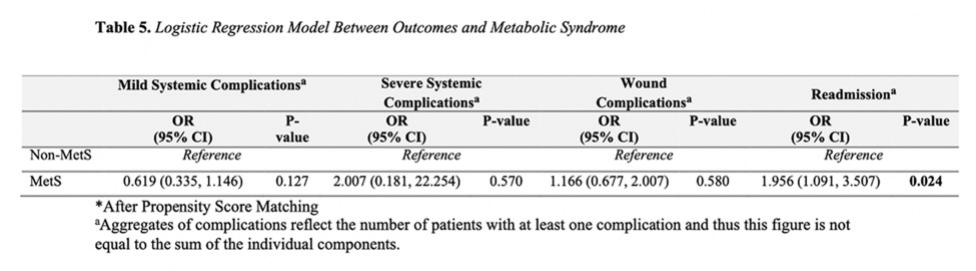# The Impact of Metabolic Syndrome on Post-Operative Outcomes in Abdominal Body Contouring: A Propensity Score-Matched Nationwide Analysis

**DOI:** 10.1093/asjof/ojae007.078

**Published:** 2024-04-12

**Authors:** Maria J Escobar-Domingo, Angelica Hernandez Alvarez, Jose Foppiani, Iulianna Taritsa, Kirsten Schuster, James Fanning, Daniela Lee, Samuel Lin, Bernard Lee

**Affiliations:** Beth Israel Deaconess Medical Center, Harvard Medical School, Boston, MA; Beth Israel Deaconess Medical Center, Harvard Medical School, Boston, MA; Beth Israel Deaconess Medical Center, Harvard Medical School, Boston, MA; Beth Israel Deaconess Medical Center, Harvard Medical School, Boston, MA; Beth Israel Deaconess Medical Center, Harvard Medical School, Boston, MA; Beth Israel Deaconess Medical Center, Harvard Medical School, Boston, MA; Beth Israel Deaconess Medical Center, Harvard Medical School, Boston, MA; Beth Israel Deaconess Medical Center, Harvard Medical School, Boston, MA; Beth Israel Deaconess Medical Center, Harvard Medical School, Boston, MA

## Abstract

**Goals/Purpose:**

Metabolic syndrome (MetS) represents cardiometabolic dysregulation, defined by hypertension, obesity, diabetes, and dyslipidemia. Its prevalence is on the rise, affecting approximately 47.3% of U.S. adults. In recent years, MetS has been associated with an elevated risk of postoperative complications. However, there remains a significant gap in our understanding of how patients with MetS fare after abdominal body contouring procedures. The objective of this study is to assess the influence of MetS on postoperative outcomes of abdominal body contouring by concurrent abdominoplasty and panniculectomy.

**Methods/Technique:**

The ACS-NSQIP database was utilized to identify patients who underwent concurrent abdominoplasty and panniculectomy procedures from 2015 to 2021. Through propensity score matching, distinct cohorts were established based on the presence of MetS, characterized by patients receiving medical interventions for diabetes mellitus and hypertension, with a body mass index exceeding 30kg/m2. Disparities among groups were assessed via unpaired T-tests and Fisher's Exact tests. Logistic regression models were constructed to evaluate the occurrence of mild and severe systemic complications, reoperation rates, and wound complications between these groups.

**Results/Complications:**

A total of 13,346 patients underwent abdominal body contouring. Following propensity score matching, 586 patients were included in the analysis, with 293 in each group (MetS vs. non-MetS). Bivariate analysis revealed a longer hospital length of stay (2.4 vs. 1.7 days; p=0.002) and higher readmission rates (11.9 vs. 6.5; p=0.031) in the MetS group in comparison to the non-MetS cohort. While surgical-related reasons for readmission were predominant across both groups, statistically significant differences were observed only for medical-related reasons (p=0.028). Logistic regression models demonstrated a statistically significant elevated likelihood of 30-day readmission in the MetS group (OR 1.96; 95% CI 1.091-3.507; p=0.024). No noteworthy disparities were observed in the rates of 30-day wound complications, mild systemic, and severe systemic complications between the groups.

**Conclusion:**

Our findings revealed no increase in postoperative wound and systemic complications among patients with MetS who underwent concurrent abdominoplasty and panniculectomy. This suggests that abdominal body contouring remains a secure option for patients with MetS. Nonetheless, the higher readmission rates and longer hospital length stays observed in patients with MetS may potentially translate to increased overall costs. Continued research is warranted to comprehensively assess the economic implications of MetS in the context of abdominal body contouring.

**Figure:**
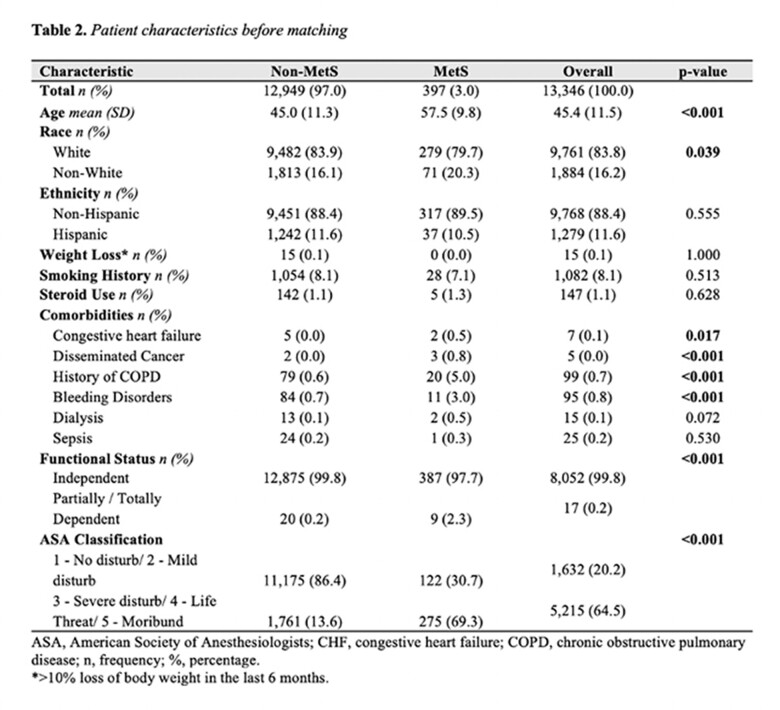

**Figure:**
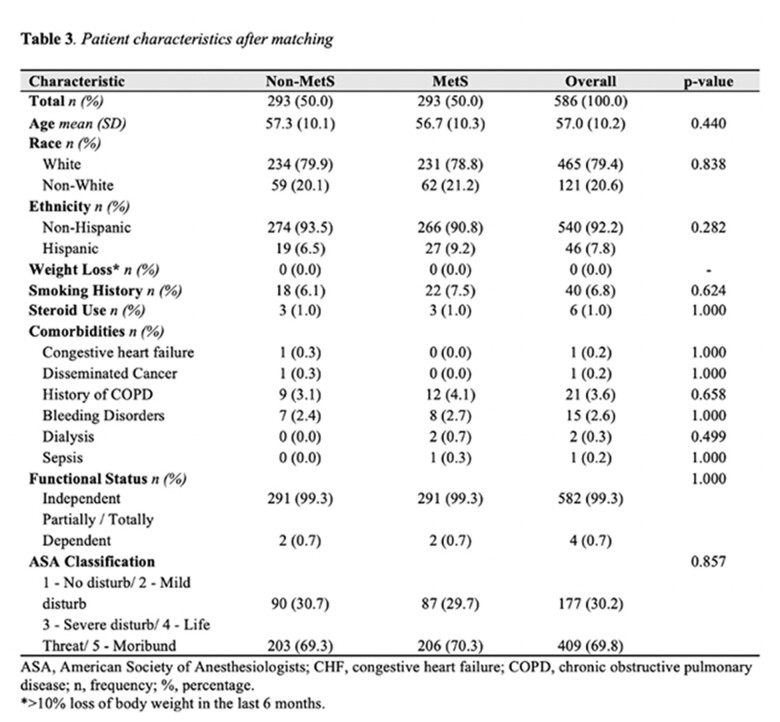

**Figure:**
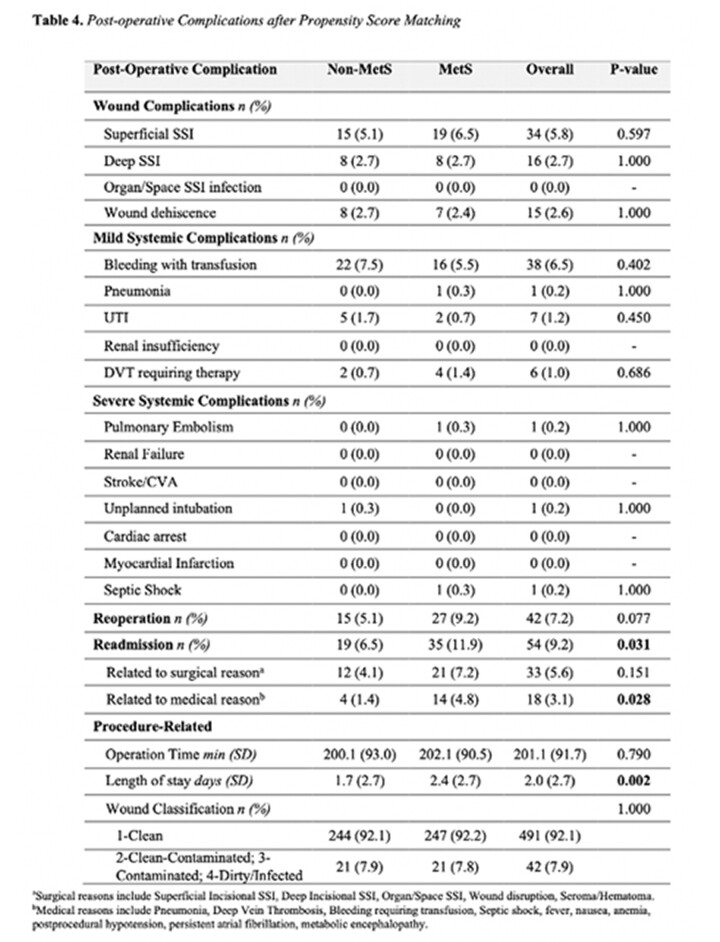

**Figure:**
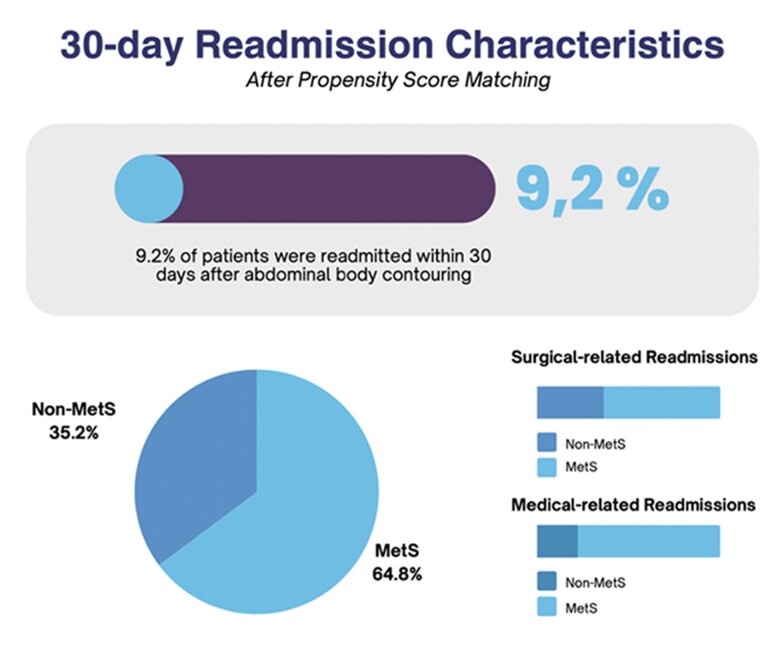

**Figure:**